# Place de la néphrolithotomie à ciel ouvert dans le traitement du calcul coralliforme: à propos d'une série de 53 patients

**DOI:** 10.11604/pamj.2019.32.110.17405

**Published:** 2019-03-08

**Authors:** Abdelilah El Alaoui, Hicham El Boté, Oussama Ziouani, Hachem El Sayegh, Ali Iken, Lounis Benslimane, Yassine Nouini

**Affiliations:** 1 Service d'Urologie A, Hôpital Ibn Sina, CHU, Rabat, Maroc

**Keywords:** Néphrolithotomie, calcul coralliforme, rein, Nephrolithotomy, coralliform stone, kidney

## Abstract

Le but de notre étude est de discuter la place de la chirurgie ouverte dans le traitement de la lithiase rénale, et notamment le coralliforme complexe, devant l'avènement de nouvelles techniques moins invasives. Nous rapportons une série de 53 calculs coralliformes colligés au sein de notre formation durant une période de 7 ans, de janvier 2011 au janvier 2018, traités par néphrolithotomie ouverte par lombotomie. La moyenne d'hospitalisation postopératoire était de 10 jours. Les suites postopératoires immédiates et précoces étaient simples chez 36 patients, 6 patients ont nécessités une transfusion sanguin, 2 ont représenté un sepsis sévère en postopératoire, 5 cas une infection de la paroi et 4 cas une fistule urinaire jugulée secondairement par un drainage endoscopique. Les calculs résiduels sont retrouvés dans 9 cas (16,9%). Ces calculs sont traités essentiellement par la lithotripsie extra corporelle. Les suites tardives étaient marquées par une atrophie rénale chez 2 patients, une récidive lithiasique chez 9 patients. Une amélioration de la clairance de créatinine chez 9 patients, et une légère aggravation chez 5 patients. La chirurgie à ciel ouvert de la lithiase rénale a de nombreuses complications, elle n'est pas recommandée en première ligne, mais il est important de reconnaître les patients chez lesquels une néphrolithotomie par voie ouverte pourrait représenter un choix valide de traitement.

## Abstract (en)

This study aims to discuss the role of open surgery in the treatment of renal lithiasis, including coralliform stones, with the advent of new less invasive techniques. We report a series of 53 cases of coralliform stones whose data were collected in our Hospital during a period of 7 years, from January 2011 to January 2018. The patients underwent open nephrolithotomy by lombotomy. The mean post-operative length of stay was 10 days. The immediate and early postoperative outcomes were simple in 36 patients, 6 patients underwent blood transfusion, 2 had severe sepsis in the postoperative period, 5 had infection of the wall and 4 had urinary fistula, secondarily managed by endoscopic drainage. Residual stones were found in 9 cases (16.9%). Stones were essentially treated by extracorporeal lithotripsy. Late outcomes were characterized by renal atrophy in 2 patients, lithiasic recurrence in 9 patients, an improvement in creatinine clearance in 9 patients and a slight worsening in 5 patients. Open surgery presents the risk of multiple complications and it is not recommended for first-line treatment of renal lithiasis. However it is important to determine which patients would benefit from open nephrolithotomy.

## Introduction

La prise en charge de la lithiase urinaire a connu de nombreux progrès. La lithotritie extracorporelle (LEC) et l'urétéroscopie souple en constitue les moyens les plus utilisés, suivie par la néphrolithotomie percutanée (NLPC), et tous ces moyens vont vers la disparition de la voie ouverte, mais cette voie s'avère parfois nécessaire devant des calculs complexes ou coralliformes. Nous essayons dans ce travail à travers l'analyse d'une série de 53 patients traités par chirurgie ouverte, et une revue de la littérature d'évaluer les résultats de cette intervention pour savoir s'il pouvait rester des indications indiscutables et si les très bons résultats en termes de *« stone free »* étaient associés à une préservation néphronique satisfaisante.

## Méthodes

**Figure 1:**
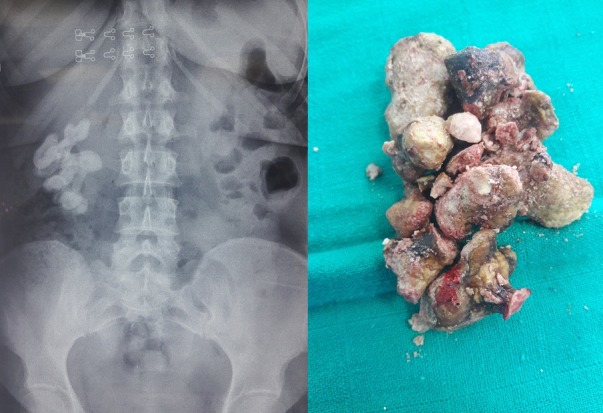
aspect d'un calcul coralliforme complet avant et après chirurgie

Nous rapportons une série de 53 calculs coralliformes colligés au sein de notre formation durant une période de 7 ans, de janvier 2011 à janvier 2018. Ces patients se répartissent en 31 femmes et 22 hommes, l'âge moyen était de 49 ans. Des antécédents de lithiase urinaire ont été notés chez 16 patients. Le délai de diagnostic moyen était de 3 ans. La symptomatologie clinique a été dominée essentiellement par la douleur puis par des manifestations infectieuses urinaires. Le Tableau 1 résume les circonstances de découvertes du coralliforme dans notre série. Une imagerie par radiographie d'abdomen sans préparation (AUSP) et uroscanner a été réalisée chez tous nos patients, ainsi que une évaluation de la fonction rénale avant et après chirurgie par mesure de la clairance de la créatinine. Le coralliforme siégeait à droite chez 28 cas, bilatérale dans 3 cas. Le calcul était radio opaque dans 46 cas. Il était complet dans 15 cas (28,3%), partiel dans 38 cas (71,6%), des pièces calicielles étaient associées dans 21 cas (39,6%). La taille moyenne des calculs était de 61 mm (40-78 mm), la Figure 1 montre un aspect d'un calcul coralliforme complet avant et après chirurgie.

**Tableau 1:** circonstances de découverte du coralliforme

Signes	Nombre de cas	Pourcentage (%)
Douleur	27	50,9
Hématurie	5	9,4
Cystite	2	3,7
Pyurie	2	3,7
Pyélonéphrite	10	18,8
Insuffisance rénale	4	7,5
Découverte fortuite	3	5,6

## Résultats

La moyenne d'hospitalisation postopératoire était de 10 jours, les suites postopératoires immédiates et précoces étaient simples chez 36 patients, 6 patients (11,3%) ont nécessités une transfusion sanguin, 2 ont représenté un sepsis sévère en postopératoire, 5 cas (9,4%) une infection de la paroi et 4 cas (7,5%) une fistule urinaire jugulée secondairement par un drainage endoscopique. Les calculs résiduels sont retrouvés dans 9 cas (16,9%). Ces calculs sont traités essentiellement par la lithotripsie extra corporelle. Les suites tardives étaient marquées par une atrophie rénale chez 2 patients, une récidive lithiasique chez 9 patients, une amélioration de la clairance de créatinine chez 9 patients, et une aggravation chez 5 patients.

## Discussion

Au cours des dernières décennies, le traitement endoscopique, la LEC et la laparoscopie ont pratiquement fait disparaître l'indication de la chirurgie lithiasique rénale par voie ouverte, y compris la néphrotomie bivalve [1], Cependant, certains cas constituent toujours un véritable challenge pour la chirurgie mini-invasive, et pour lesquels la chirurgie ouverte reste l'option de choix. selon les recommandations de l'Association européenne d'urologie (EAU guidelines), on trouve que dans les calculs complexes y a compris les calculs coralliforme partiel ou complet, le traitement de premier ligne est la NLPC ou la chirurgie combinée entre la NLPC et l'urétéroscopie souple, Cependant, si les approches percutanées ne sont pas susceptibles de réussir, ou si plusieurs approches endo urologiques ont été effectuées sans succès; la chirurgie ouverte ou laparoscopique peut être une option de traitement valide (Tableau 2) [2]. Assimos *et al* [3], ont comparé la néphrolitothomie ouverte et la NLPC avec ou sans LEC complémentaire. Un volume lithiasique élevé était associé à une multiplication des procédures, plus d'hospitalisations et un coût global des soins plus élevé pour les patients opérés par NLPC. Les résultats de la NLPC, restent inférieurs à ceux de la chirurgie ouverte pour l'absence de fragment résiduel dans la chirurgie du calcul coralliforme complexe. Les taux retrouvés dans la littérature varient de 49 à 90 % selon les séries [3,4], contre des taux variant de 82,1 à 100 % selon les séries pour la chirurgie ouverte [3,5-7].

**Tableau 2:** recommandations de l’association européenne d’urologie (EAU guidelines2018)

Recommendations	Strength rating
Offer laparoscopic or open surgical stone removal in rare cases in which shock wave lithotripsy (SWL), (flexible) ureterorenoscopy and percutaneous nephrolithotomy fail, or are unlikely to be successful	Strong
Perform surgery laparoscopically before proceeding to open surgery	Strong
For ureterolithotomy, perform laparoscopy for large impacted stones when endoscopic lithotripsy or SWL has failed or is contraindicated	Strong

Dans notre série, l'absence de fragment résiduel a été observée chez 83,1% des patients, ce qui rejoint les données de la littérature. Ces très bons résultats sur le volume lithiasique et le stone free, sont obtenus au prix d'une morbidité non négligeable tant pour les complications postopératoires que pour la préservation néphronique. Snyder et Smith [5], comparant l'extraction percutanée avec la néphrolitothomie ouverte pour calculs coralliformes, ont rapporté un bénéfice de la voie percutanée pour la durée opératoire, le taux de transfusion et le temps nécessaire aux patients pour reprendre une activité quotidienne normale. Lunardi *et al* [8], ont présenté une des plus grandes séries de néphrolithotomie bivalve par lombotomie (cohorte de 2^6^ patients), Le taux de patients sans résidu lithiasique suite à la procédure était de 92%, une diminution négligeable des chiffres de clairance de la créatininémie a été observée chez 4 patients (16%) et une amélioration chez 10 patients avec augmentation de la clairance de 12 mL/min/m^2^ en moyenne, la morbidité était relativement faible, puisque seulement 2 patients ont présenté une complication de grade III (deux abcès de paroi dont un suivi d'une fistule uro-cutanée résolutive avec traitement médical). La morbidité per opératoire dans notre série était relativement faible puisque 26,4% des patients ont fait des complications de grade II et III selon la classification de Clavien: 8 complications de grade II (6 déglobulisations nécessitant une transfusion, et 2 sepsis), 2 complications de grade IIIA (2 abcès de parois traités par incision au lit du patient et antibiothérapie) et 4 complication de grade IIIB (fistule urinaire traitée avec succès par drainage prolongé par sonde JJ). On a noté une amélioration de la clairance de créatinine chez 9 patients, et une légère aggravation chez 5 patients.

## Conclusion

Le calcul coralliforme est un calcul assez fréquent, il est grave par son retentissement sur le rein, l'infection urinaire associé et le risque de récidive important. Son traitement par voie ouverte a de nombreuses complications, elle n'est pas recommandée en première ligne, mais il est important de reconnaître les patients chez lesquels une néphrolithotomie par voie ouverte pourrait représenter un choix valide de traitement.

### Etat des connaissances actuelles sur le sujet

- Les progrès de l'endo-urologie ont considérablement réduit les indications de la chirurgie ouverte dans le traitement du calcul rénal coralliforme;
- La chirurgie à ciel ouvert ou laparoscopique reste recommandée pour le traitement des calculs coralliformes dont le nombre prévisible d'accès percutanés paraît déraisonnable.

### Contribution de notre étude à la connaissance

- Discuter la place de la chirurgie ouverte dans le traitement de la lithiase rénale, et notamment le coralliforme complexe, devant l'avènement de ces techniques moins invasives.

## Conflits des intérêts

Les auteurs ne déclarent aucun conflit d'intérêts.
